# Case report: Detecting giant cell arteritis in [^68^Ga]Ga-DOTA-Siglec-9-PET/CT

**DOI:** 10.3389/fimmu.2024.1501790

**Published:** 2024-12-16

**Authors:** Simon M. Petzinna, Jim Küppers, Benedikt Schemmer, Anna L. Kernder, Claus-Jürgen Bauer, Leon von der Emde, Babak Salam, Jörg H. W. Distler, Anja Winklbauer, Markus Essler, Valentin S. Schäfer

**Affiliations:** ^1^ Department of Rheumatology and Clinical Immunology, Clinic of Internal Medicine III, University Hospital Bonn, Bonn, Germany; ^2^ Department of Nuclear Medicine, University Hospital Bonn, Bonn, Germany; ^3^ Clinic for Rheumatology, University Hospital Düsseldorf, Medical Faculty of Heinrich-Heine-University, Düsseldorf, Germany; ^4^ Hiller Research Center, University Hospital Düsseldorf, Medical Faculty of Heinrich-Heine-University, Düsseldorf, Germany; ^5^ Department of Ophthalmology, University Hospital Bonn, Bonn, Germany; ^6^ Department of Radiology, University Hospital Bonn, Bonn, Germany

**Keywords:** giant cell arteritis, vasculitis, inflammation, imaging, biomarker, positron emission tomography-computed tomography

## Abstract

**Objectives:**

This study aimed to evaluate the diagnostic utility of [^68^Ga]Ga-DOTA-Siglec-9 positron emission tomography-computed tomography (PET/CT) in assessing disease activity in a patient experiencing a relapse of giant cell arteritis (GCA).

**Case presentation:**

A 90-year-old male patient with GCA, diagnosed in 2018, was enrolled. Demographic data, disease history, and laboratory parameters, including soluble VAP-1 (sVAP-1) levels, were recorded. The patient underwent a [^68^Ga]Ga-DOTA-Siglec-9 PET/CT scan. Additional imaging assessments included vascular ultrasound of the superficial temporal arteries, their branches, and the facial, axillary, subclavian, carotid, and vertebral arteries, along with magnetic resonance imaging (MRI) of the aorta.

The patient’s sVAP-1 level was 284 ng/ml compared to 123 ng/ml in the control group (SD ± 55). The [^68^Ga]Ga-DOTA-Siglec-9 PET/CT scan revealed increased tracer uptake (SUVmax) in the subclavian artery (2.5), aortic arch (2.9), and heart (2.9). Notably, the increased uptake in the descending aorta (3.5) abruptly diminished to 2.2 when passing the diaphragm, with no changes in vessel caliber observed in CT. The injection of [^68^Ga]Ga-DOTA-Siglec-9 was well tolerated. Aortic MRI revealed no signs of inflammatory involvement.

**Conclusions:**

This study introduces the first application of [^68^Ga]Ga-DOTA-Siglec-9 PET/CT in a patient with GCA experiencing a relapse, revealing enhanced tracer uptake in the subclavian artery and aortic arch with a localized and abrupt reduction, absent in conventional imaging. These findings suggest that [^68^Ga]Ga-DOTA-Siglec-9 PET/CT has significant potential for precise, inflammation-specific detection of affected vascular tissue in GCA during relapse.

## Introduction

Giant cell arteritis (GCA) is an immune-mediated vasculitis affecting large and medium-sized vessels. It can lead to vascular changes and occlusion due to severe vascular inflammation, neoangiogenesis, and remodeling. Vascular ultrasound of the temporal and axillary arteries has replaced temporal artery biopsy as the primary diagnostic tool for GCA in most centers (1, 2). However, ultrasound is limited in assessing the whole aorta, which is concerning given that up to 65% of GCA patients may exhibit aortic involvement (3, 4). Consequently, positron emission tomography-computed tomography (PET/CT) with [^18^F]fluordeoxyglucose (FDG) has become instrumental in diagnosing systemic manifestations of GCA (5). [^18^F]FDG-PET-CT may also aid in monitoring disease activity in GCA, as FDG uptake generally decreases with treatment (6, 7). However, interpreting follow-up scans is challenging due to increased glucose metabolism associated with vascular remodeling (6, 7). Persistent elevated FDG uptake, seen in up to 80% of patients, limits its specificity in detecting active inflammation (6–8). Similarly, persistence of disease-specific patterns of GCA complicate the use of CT (9) and magnetic resonance imaging (MRI) (10) in assessing current disease activity or detecting relapses in GCA.

The introduction of new, more inflammation-specific radiotracers, such as [^68^Ga]Ga-DOTA-Siglec-9, promises to improve the assessment of inflammatory vascular diseases. Early studies in animals and humans have demonstrated its safety, tolerability, and potential efficacy in identifying inflammation (11–13).

Sialic acid-binding immunoglobulin-like lectin-9 (Siglec-9) serves as a leukocyte ligand for vascular adhesion protein 1 (VAP-1) (11). VAP-1, a product of the amine oxidase copper-containing 3 gene, exists both as a soluble form (sVAP-1) with enzymatic activity and as a membrane-bound endothelial adhesion molecule (14). Under physiological conditions, VAP-1 resides in intracellular vesicles within various cell types, including endothelial cells. Upon inflammatory stimuli such as tumor necrosis factor-α, interferon-γ, and interleukin-1β, VAP-1 is mobilized to the endothelial cell surface, enabling its interaction with Siglec-9 on granulocytes and monocytes (12). This interaction triggers oxidative deamination, resulting in cytotoxic damage to endothelial cells and driving inflammatory response (14, 15). It promotes the synthesis of key endothelial adhesion molecules, including ICAM-1, Mad-CAM-1, E-selectin, and P-selectin. Additionally, the secretion of the chemokine CXCL8, activation of transcription factors such as NF-κB, and expression of matrix metalloproteinases are enhanced, facilitating leukocyte rolling, tethering, and migration (14).

The selective upregulation of VAP-1 during vascular inflammation renders [^68^Ga]Ga-DOTA-Siglec-9-PET/CT particularly promising for the assessment of GCA (16). In this study, we are the first to evaluate the diagnostic utility of [^68^Ga]Ga-DOTA-Siglec-9-PET/CT for assessing disease activity in a patient experiencing a GCA relapse.

## Case description

### Clinical and laboratory assessment

A 90-year-old male patient with GCA, initially diagnosed in August 2018 and fulfilling the American college of rheumatology (ACR)/European league against rheumatism (EULAR) classification criteria (2), was recruited at the Department of Rheumatology (University Hospital Bonn, Germany) in November 2023. He reported the recurrence of GCA symptoms, which included bitemporal headaches and night sweats. At the time of his scan, he was receiving 15 mg of methotrexate subcutaneously q1w and 2 mg of prednisolone q1d. His symptoms had re-emerged when the prednisolone dosage was reduced below 4 mg/day. Laboratory analysis revealed an elevated C-reactive protein (CRP) level at 21 mg/l and an erythrocyte sedimentation rate of 47 mm/hr and 81 mm/2hrs.

An enzyme-linked immunosorbent assay (ELISA) was performed to assess the sVAP-1 level (Human VAP-1 Quantikine ELISA Kit, R&D systems, Minneapolis, USA). For control, we analyzed the sVAP-1 levels of 15 healthy individuals. As samples generated values higher than the highest standard, we further diluted with calibrator diluent (1:200). The patient’s sVAP-1 level was 284 ng/ml, higher than the control group’s levels (mean 123 ng/ml, median 117 ng/ml, SD ± 55, min. 59 ng/ml, max. 241 ng/ml) (**Supplementary Figure 1**).

### [^68^Ga]Ga-DOTA-Siglec-9-PET/CT

**Figure 1:**
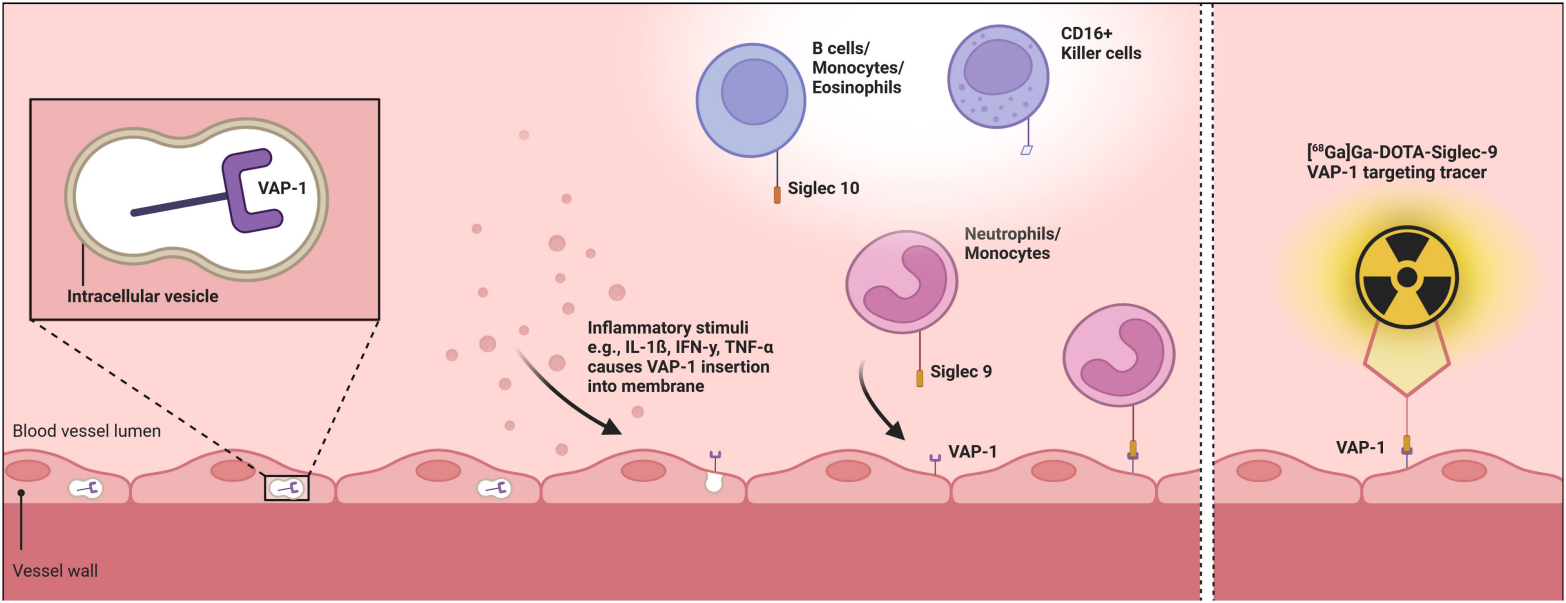
Pathophysiological role of VAP-1 and its radioactive labeling by the [^68^Ga]Ga-DOTA-Siglec-9 tracer. The first part of the figure schematically illustrates the endothelial translocation of VAP-1 from intracellular vesicles following inflammatory stimuli, along with the subsequent binding of neutrophils and monocytes via the Siglec-9 ligand, while the second part depicts the [^68^Ga]Ga-DOTA-Siglec-9 radiotracer bound to endothelially expressed VAP-1. Created with BioRender.com. VAP-1, vascular adhesion protein 1; Siglec 9, sialic acid-binding immunoglobulin-like lectin-9.

Radiosynthesis of [^68^Ga]Ga-DOTA-Siglec-9 with 1.55 GBq gallium-68 revealed a decay corrected radiochemical yield of 95.1% (77.9% non-decay corrected radiochemical yield). A volume activity of 130.0 MBq/mL and an apparent molar activity of 21.0 MBq/nmol were obtained. Radiochemical purity was >98%. The patient underwent a [^68^Ga]Ga-DOTA-Siglec-9 PET-CT scan with an injection of 120 MBq [^68^Ga]Ga-DOTA-Siglec-9. Fifty-one minutes post-injection, we conducted a low-dose CT for attenuation correction and a whole-body PET scan (one minute/bed). The decision regarding the 51-minute post-injection delay was guided by prior research (13), from which we determined a minimum delay of 40 minutes post-injection. The protocol is available with the full text of this article online (**Figure 1**).

**Figure 2:**
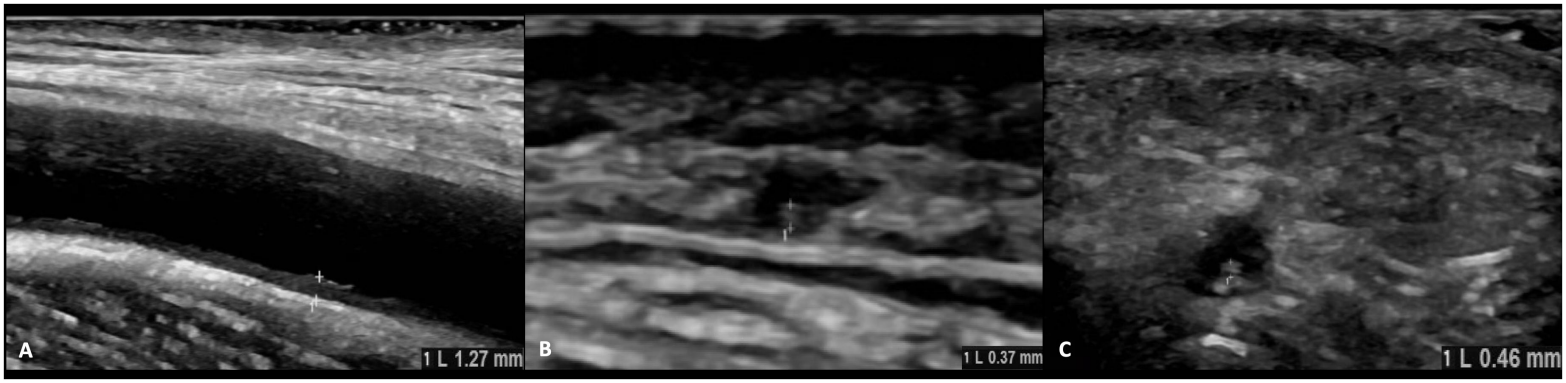
Vascular ultrasound and pathological intima-media thickness. This figure presents the outcomes of a detailed ultrasound evaluation of the superficial temporal arteries along with their branches, and the facial, axillary, brachial, subclavian, and carotid arteries, displaying those with pathological intima-media thickness. The right axillary artery (1.23 mm), right frontal lobe of the temporal artery (0.37 mm), and right facial artery (0.46 mm) surpass the established threshold values with no changes in the extent of vascular involvement in comparison to previous examinations. **(A)** right axillary artery, **(B)** right ramus frontalis, **(C)** right facial artery.

[^68^Ga]Ga-DOTA-Siglec-9 PET scan revealed increased tracer uptake (SUVmax) in the subclavian artery (2.5), aortic arch (2.9), and heart (2.9), in contrast to an uptake of 1.5 in the liver (**Figure 2**). Notably, an increased uptake in the descending aorta (3.5) abruptly diminished to (2.2) while passing the diaphragm but before reaching the cealic trunk (2 cm subdiaphragmatic), with only minimal changes in vessel caliber (29x30mm) and no intermediate branching vessels. The injection of [^68^Ga]Ga-DOTA-Siglec-9 was well tolerated.

### Further imaging techniques

The patient underwent a comprehensive ultrasound examination of the common superficial temporal arteries with their frontal and parietal branches, as well as the facial, axillary, brachial, subclavian, and carotid arteries. The examination was conducted using a GE LOGIQ e10 (2021) ultrasound machine. Measurement of the highest intima-media thickness (IMT) of the arteries was conducted in B-mode at the point of greatest extent of the distal vessel and compared to established cut-off values (17). Outcome Measures in Rheumatology (OMERACT) ultrasound score for monitoring disease activity in GCA was calculated (18). Values were compared to vascular ultrasound results from the previous visit six weeks before.

**Figure 3:**
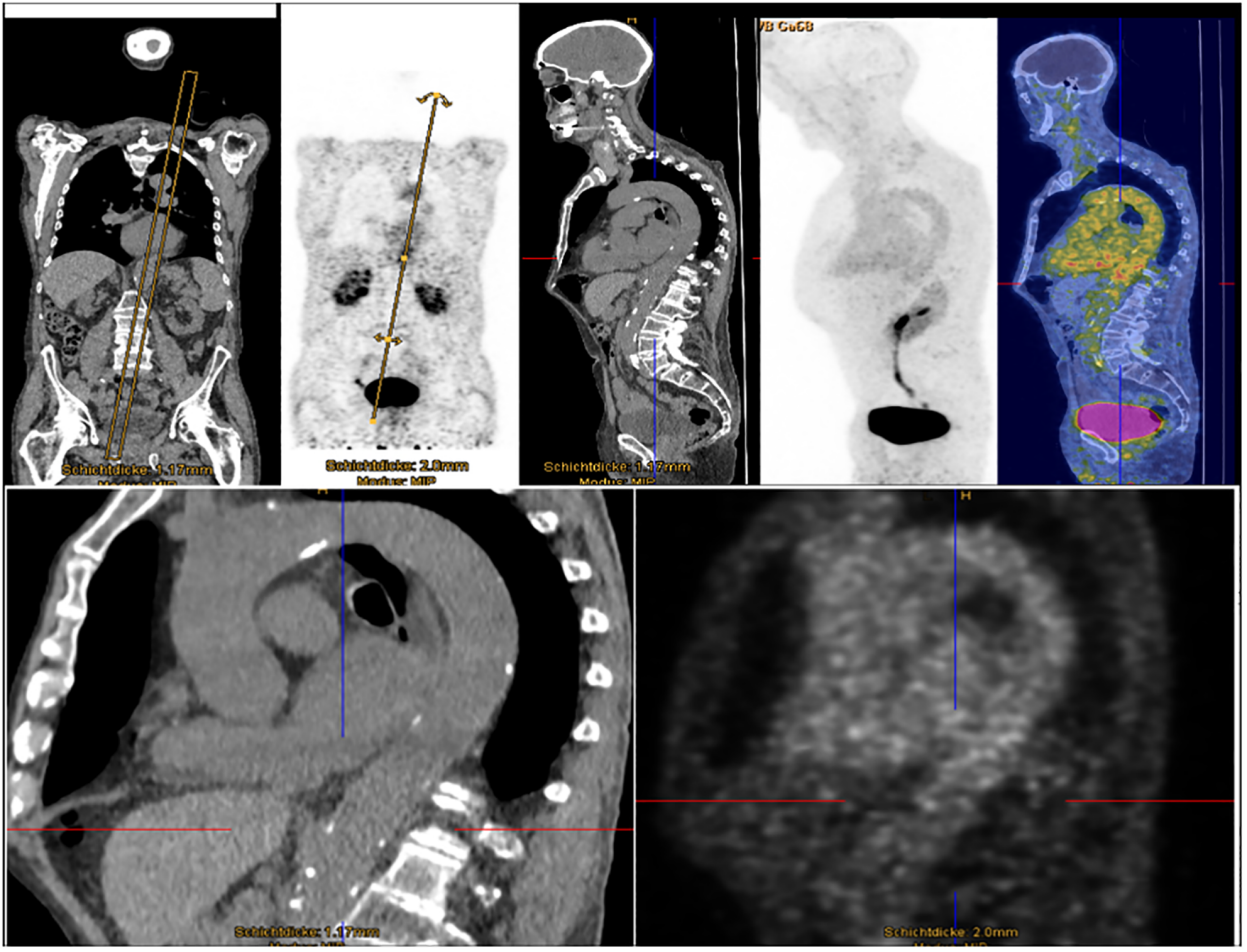
Enhanced [^68^Ga]Ga-DOTA-Siglec-9 uptake in aortic and subclavian regions. Whole-body PET images after intravenous injection of 120 MBq of [^68^Ga]Ga-DOTA-Siglec-9. Distribution of [^68^Ga]Ga-DOTA-Siglec-9 51 min after injection, based on imaging for one minute per bed position, revealed increased uptake in projection on the aortic and subclavian vessels and the heart. The increased uptake in the descending aorta diminishes abruptly while passing the diaphragm, without any caliber jump in the CT images.

The IMT measurements for the right axillary artery (1.23 mm), frontal branch (0.37 mm), and facial artery (0.46 mm) surpassed the established cut-off values (17) (**Figure 3**), displaying no changes in the degree of vascular involvement compared to the results of the last visit (data not shown). No other arteries examined, exhibited pathological IMT (data not shown). The OMERACT ultrasound score registered at 0.95 at the time of the scan, showing a slight decrease from 1.0 recorded six weeks before.

Additionally, a comprehensive ophthalmological assessment was conducted, encompassing evaluations of best-corrected visual acuity, intraocular pressure, and optical coherence tomography (angiography). The findings for both eyes were consistent with normal age-related conditions, and ocular complications related to GCA were ruled out at the time of examination (**Supplementary Figure 2**).

Moreover, MRI assessment of the aorta was conducted utilizing a 3-Tesla system (Ingenia 3.0T, Philips Healthcare, Best, The Netherlands). A 18-channel body array coil was employed for signal reception. The acquisition protocol included a coronary balanced gradient echo sequence, non-Cartesian axial T2 SPAIR, axial diffusion-weighted imaging (DWI) with b-value of 800 s/mm², and axial steady-state free precession. Aortal MRI revealed no signs of inflammatory involvement of GCA (**Supplementary Figure 3**).

## Discussion

This study aimed to evaluate the diagnostic utility of the radiotracer [^68^Ga]Ga-DOTA-Siglec-9 for the assessment of vascular inflammation in GCA relapse.

While vascular ultrasound can be valuable in monitoring the disease and detecting relapses (19, 20) typically showing a reduction in IMT over the course of the disease, a significant limitation is its inability to comprehensively assess the entire aorta. This limitation is critical given that aortitis can lead to dilation, aneurysm, or dissection. In contrast, [^18^F]FDG-PET/CT offers a comprehensive assessment of all major arteries involved. However, persistent elevated uptake due to vascular remodeling is frequently observed even in remission, with reported diagnostic sensitivity and specificity at 77% and 71%, respectively (8).

Our pioneering, exploratory case report represents the first application of [^68^Ga]Ga-DOTA-Siglec-9 PET/CT in the context of GCA relapse, revealing enhanced tracer uptake in the subclavian artery and aortic arch with a localized and abrupt reduction. Intriguingly, these findings were not mirrored in aortic MRI imaging, and neither vascular ultrasound nor ophthalmological evaluations identified any new pathological changes. This discrepancy highlights a substantial diagnostic gap, underscoring the limitations of current diagnostic strategies in accurately identifying GCA relapses.

This study introduces [^68^Ga]Ga-DOTA-Siglec-9-PET/CT as a novel imaging approach for the identification of inflammation in the vascular system during GCA relapse. The affinity for inflammatory processes, driven by VAP-1, offers a promising avenue to refine diagnostic protocols for evaluating GCA activity. Our data also confirm the safety and tolerability of [^68^Ga]Ga-DOTA-Siglec-9 imaging, aligning with prior research (12, 13). Notably, [^68^Ga]Ga-DOTA-Siglec-9-PET demonstrated a lower radiation dose compared to [^18^F]FDG-PET (approximately 3 mSv vs. up to 15 mSv), making it especially appealing for reducing radiation exposure (13).

The detection of an elevated sVAP-1 level in our GCA patient compared to healthy controls suggest its potential as a biomarker for GCA. Elevated sVAP-1 levels may reflect heightened disease activity, as previously shown by the correlation between sVAP-1 concentrations and membrane-bound VAP-1 activity (14). However, sVAP-1’s role in GCA pathogenesis requires further validation, including the influence of matrix metalloproteinases, which contribute to endothelial cleaving. In this context, larger studies are needed to improve generalizability.

In summary, our study suggests that [^68^Ga]Ga-DOTA-Siglec-9-PET/CT could complement established imaging techniques for assessing GCA disease activity, potentially addressing limitations of traditional diagnostics. By targeting VAP-1, this method may allow precise localization and evaluation of systemic vascular inflammation, potentially reducing reliance on inconclusive multimodal approaches and enhancing diagnostic strategies and therapeutic management in GCA.

Several limitations should be noted: the single-patient design of our case report restricts the generalizability of imaging and biomarker assessments. A comparative [^18^F]FDG-PET/CT scan could elucidate the specific advantages and limitations of [^68^Ga]Ga-DOTA-Siglec-9 in evaluating GCA. Additionally, assessing diagnostic utility in newly diagnosed patients would be insightful. Future studies should further aim to establish standardized imaging protocols and include larger patient cohorts to reinforce its diagnostic utility in GCA management.

## Data Availability

The original contributions presented in the study are included in the article/**Supplementary Material**. Further inquiries can be directed to the corresponding author.
