# Supplementary material for: Case report: Detecting giant cell arteritis in [68Ga]Ga-DOTA-Siglec-9-PET/CT
Source: Front Immunol. 2024 Dec 16;15:1501790. doi: 10.3389/fimmu.2024.1501790 (PMC11701584; doi:10.3389/fimmu.2024.1501790)
Supplement: Supplementary file 1 [file DataSheet1.docx]

**Supplemental Figures**


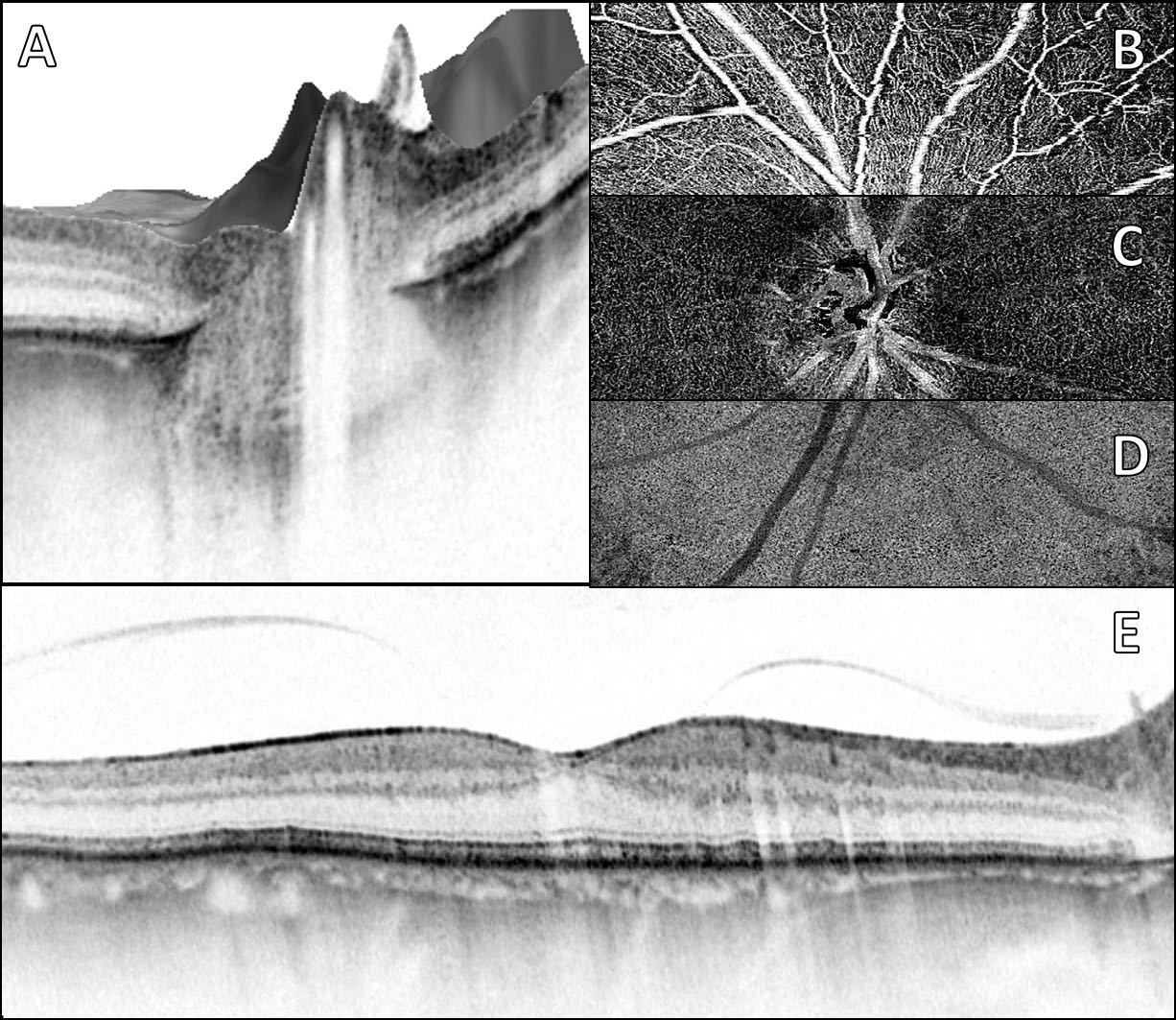


sFigure 1: Multimodal retinal imaging of the optic nerve and macula

Best-corrected visual acuity was recorded at 1.0 for both eyes, with intraocular pressure measured at 14 mmHg for the right eye and 16 mmHg for the left eye. Panel A displays a three-dimensional view of the optic nerve head using Optical Coherence Tomography (OCT). Panels B to D illustrate OCT angiography images that showcase the superficial (B), deep vascular plexus (C), and the choriocapillaris (D) surrounding the optic nerve head. In this instance, there are no signs of perfusion abnormalities in the optic nerve head, indicating intact blood supply. Panel E depicts an OCT scan of the macula through the fovea, where isolated age-related changes suggestive of early age-related macular degeneration are observed. Nonetheless, the retinal layering and foveal contour appear normal. Overall, the findings for both eyes are consistent with normal age-related conditions, and ocular complications related to giant cell arteritis were ruled out at the time of examination.


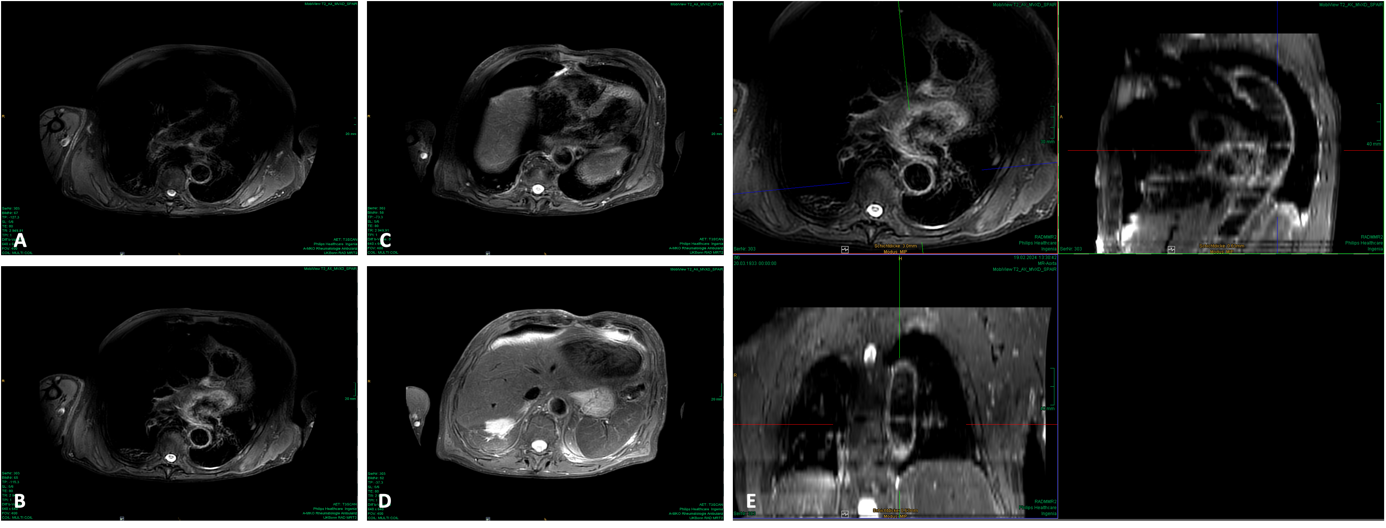


sFigure 2: Images from an magnetic resonance imaging of the aorta.

Representative axial images at various levels of the descending aorta to below the diaphragmatic transition (A-D) do not reveal aortitis-typical changes in the aortic vessel wall. Similarly, in the reconstructed images (E), no differences in vessel wall structure between the supradiaphragmatic and infradiaphragmatic aorta are apparent.


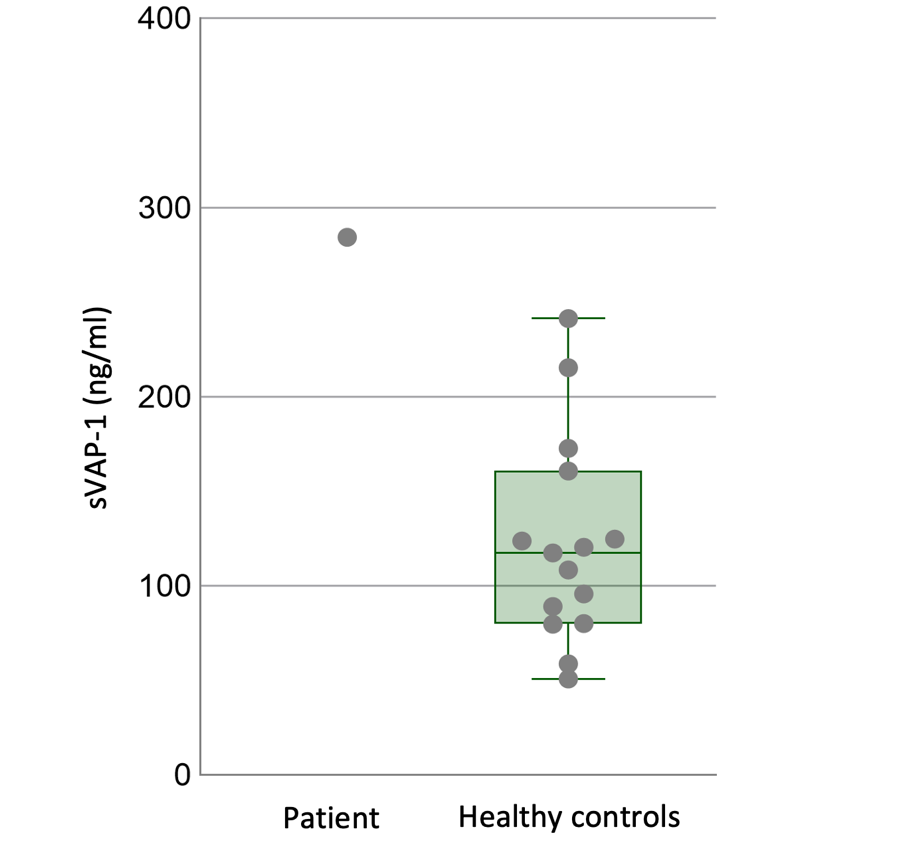


sFigure 3: Comparative analysis of soluble vascular adhesion protein 1 levels

This figure depicts the outcomes of a soluble vascular adhesion protein-1 level analysis, conducted with ELISA, comparing our case report patient against a control group of 15 healthy individuals (mean age 31.5 years, female: 8, male: 7). The serum VAP-1 concentration in the case report patient was identified at 284 ng/ml, higher than the control group's, which displayed a median of 117 ng/ml, a 25% quantile of 85 ng/ml, a 75% quantile of 143 ng/ml, with a minimum of 59 ng/ml, a maximum of 241 ng/ml and a standard deviation of 55 ng/ml. Abbrv.: sVAP-1: soluble vascular adhesion protein 1.
